# Combination of interventional adenovirus-p53 introduction and ultrasonic irradiation in the treatment of liver cancer

**DOI:** 10.3892/ol.2014.2811

**Published:** 2014-12-18

**Authors:** JIN-SONG QI, WEN-HUI WANG, FEN-QIANG LI

**Affiliations:** 1 Department of Interventional Radiology, The First Affiliated Hospital of Xinxiang Medical University, Xinxiang, Henan 453100, P.R. China; 2 Department of Interventional Medicine, The First Hospital of Lanzhou University, Lanzhouu, Gansu 730000, P.R. China

**Keywords:** adenovirus-p53, liver cancer, interventional introduction, ultrasonic irradiation

## Abstract

The aim of the present study was to investigate the effect of the combination of interventional adenovirus-p53 (Ad-p53) introduction and ultrasonic irradiation (CIAIUI) treatment for liver cancer, including evaluating the Ad-p53 transfection efficiency and the impact of the p53 gene on vascular endothelial growth factor (VEGF) and matrix metalloprotein 2 (MMP2) protein expression levels. Ad-p53 was arterially infused into the hepatic carcinoma via the interventional introduction of the hepatic tumor-bearing artery (IIHTBA) or the CIAIUI. Serum VEGF levels were determined by performing an enzyme-linked immunosorbent assay; immunohistochemical analysis was used to identify the expression levels of intratumoral p53, MMP2 and VEGF; and western blot analysis was used to determine the impact of different Ad-p53 administration methods on the expression of wild-type p53. The wild-type p53 expression level was significantly higher in the p53-treated group compared with the control group, and the p53 expression level in the CIAIUI group was significantly higher compared with the non-irradiation group. The CIAIUI could significantly reduce the serum VEGF levels. The two delivery methods caused a reduction in the intratumoral VEGF and MMP2 expression levels, and the effects of CIAIUI were most obvious. Ad-p53 infusion via IIHTBA promoted the protein expression levels of p53, however, it inhibited the protein expression levels of MMP2 and VEGF, indirectly indicating that the gene may inhibit the growth of liver cancer. Therefore, CIAIUI therapy exhibited an overall improved therapeutic effect compared with the more simple IIHTBA therapy.

## Introduction

Liver cancer is one of the most common types of malignant tumor in humans; its incidence is the fifth highest amongst the malignant tumors (1,2). China has a particularly high prevalence of liver cancer and the number of resulting annual mortalities account for ~45% of the global mortality rate due to liver cancer. The traditional treatment strategy for liver cancer includes surgery, radiotherapy and chemotherapy. Surgery has the highest potential for reducing tumor burden; however, numerous patients are already in the middle or advanced stages of the disease when they enroll in treatment, and are therefore past the optimum stage for undergoing surgery. Although systemic chemotherapy may be used in the treatment of advanced liver cancer, patients often find the side effects difficult to tolerate. The total effective dose of liver cancer radiotherapy is 60 Gy, however, healthy liver tissue can only tolerate a limit of 40 Gy and, therefore, radiotherapy cannot be used as the conventional treatment strategy for liver cancer. Thus, there is a requirement for the development of radical treatment strategies for liver cancer.

Transcatheter arterial chemotherapy (TAC) has become an important method in the treatment of liver cancer patients in the middle or advanced stages of the disease, as it is minimally invasive, uses high local concentrations of chemotherapeutic agents and exhibits small systemic side effects. TAC therapy is based on the evidence that blood is primarily supplied to healthy liver tissues via the hepatic portal vein, while the hepatic artery accounts for >80% of the blood supplied to liver cancer (3–5).

As research into oncobiology has increased, the development of tumor gene therapy has broadened the treatment prospects for cancer patients (6). Liver cancer is characterized by the type of the gene mutation present; the majority of cases of liver cancer are caused by the deletion of the p53 tumor suppressor gene (also known as the wild-type p53 gene, wt-p53). It is reported that ~50% of cases of human cancer are associated with the p53 gene mutation (i.e., mutated-type p53 gene), and cannot express normally functioning p53 (7,8), resulting in malignancy. Previous studies have identified that the introduction of wt-p53 into tumor cells may result in cells which are able to effectively perform p53 functions, such as proliferation inhibition, induction of apoptosis, inhibition of angiogenesis, delayed metastasis and increased sensitivity to chemotherapeutic agents; thus, wt-p53 itself may act as a therapeutic agent against the cancer (9). The transfection efficiency of wt-p53 into tumor cells is associated with the local intratumoral concentration of the p53 gene; the higher the concentration of the p53 gene, the higher the transfection efficiency and the greater efficacy towards the tumor cells (10). This p53-based gene therapy has evolved from basic research to clinical practice; for example, the Ad-p53 Gendicine has been approved as part of biological cancer therapy in China (11). However, how to reduce the risks of gene therapy in clinical practice, improve the transfection efficiency of the exogenous p53 gene in liver tumor cells, increase Ad-p53 target specificity, reduce the immune response and increase the efficiency of gene therapy require further investigation.

Progress has occurred in the field of ultrasound-mediated gene transfection, and sufficient gene transfection efficiency has been achieved (12,13). The effective transfection efficiency and expression levels of the exogenous genes in tumor cells is the key to the success of gene therapy, and changes in the cell membrane and capillary wall permeability are prerequisites of gene transfection. The microbubble ultrasound contrast agents may reduce the ultrasonic cavitation threshold, enhance the ultrasonic cavitation effects, and further improve cell membrane and capillary wall permeability, therefore, significantly enhancing the transfection efficiency and expression levels of the exogenous gene in the tumor cells (14). The ultrasound contrast agent SonoVue^®^ is a type of sonicated albumin microbubble; the albumin coat of the microbubble may maintain the original biological characteristics, combining with the proteins and antisense oligonucleotides (15,16), therefore, it may be used as a vector in gene therapy. If this microbubble could be combined with target therapeutic genes, and if ultrasonic irradiation could be used to destruct the bubble when the therapeutic gene was delivered to the lesions via blood flow, then the therapeutic gene and microbubble coat would be locally released into the tumor tissue spaces through the permeability-increased blood vessel walls. In addition, the shock wave generated by the microbubble rupture may promote the entry of the gene into the surrounding tumor tissues. Rat experiments conducted by Skyba *et al* (17) and Shohet *et al* (18) demonstrated that, following the adhesion of the gene to the ultrasound contrast agent, the ultrasonic irradiation enhanced the gene transfection efficiency.

The present study aimed to explore the method of combined interventional Ad-p53 introduction and ultrasound irradiation (CIAIUI), using the ultrasonic microbubble agent SonoVue^®^, for infusion into liver lesions via the hepatic artery. The present study hypothesized that the addition of ultrasound irradiation would improve the intratumoral transfection efficiency of the Ad-p53 gene. Additionally, the study aimed to observe the inhibition efficiency of vascular endothelial growth factor (VEGF) and matrix metalloprotein 2 (MMP-2) and explore the underlying molecular mechanism of this process.

## Materials and methods

### Animals

Fifteen male and fifteen female chinchilla rabbits (weight, 2.5–3.0 kg) were provided by the Experimental Animal Center of the Lanzhou Institute of Biological Products (Lanzhou, China) The VX2 tumor line was provided by Professor Hongxin Zhang (Department of Interventional Radiology, the Fourth Military Medical University, Xi’an, China). This study was approved by the ethics committee of The First Affiliated Hospital of Xinxiang Medical University (Xinxiang, China).

### Animal model of liver cancer

The frozen VX2 tumor cell line was recovered and the tumor cell concentration was adjusted to 10^9^ cells/l. The prepared tumor cell suspension (dose, 0.5 ml) was inoculated into the rabbit hind lateral muscle and two weeks later the rabbits had developed sufficient tumors. The tumor-bearing rabbits were anesthetized with 50 mg/kg pentobarbital sodium and the tumor was resected under sterile conditions, cut into three 1-mm sections, and suspended in saline to create a tumor tissue block suspension at a concentration of 5×10^10^ sections/l. Following general anesthesia with 50 mg/kg pentobarital sodium, the rabbits were fixed to anatomical plates, the skin of the upper abdomen was sterilely prepared and ~3-cm longitudinal incisions were made layer by layer to expose and fix the liver. An inoculation needle was used to inject 1-ml tumor tissue block suspension into the rabbit liver under direct vision with a needle depth of ~2 cm. Gauze was used for local hemostasis compression for 2 min and the abdominal wall was sutured layer by layer. Three days later, each rabbit model was intramuscularly injected with 400,000 U/day penicillin to prevent infection, and the tumor growth was ultrasonically monitored on days 7, 10 and 14 following inoculation.

### Ad-p53 infusion

The rabbit models that survived for 15 days were randomly divided into three groups (n=10 per group). The rabbits were anesthetized with 50 mg/kg pentobarbital sodium, fixed and, under the sterile conditions, the skin and the subcutaneous tissues of the groin area were incised layer by layer until the femoral artery was exposed. A radial artery puncture needle was used to puncture the femoral artery and, under the guidance of Coroskop TOP digital subtraction machine (Siemens AG, Munich, Germany) and with the aid of a microwire, a microcatheter was placed in the tumor-bearing artery. In group one (control group), 10 ml saline was infused through the catheter; in group two (p53 group), 10 ml Ad-p53 was infused through the microcatheter [(1×10^11^ viral particles (vp); SiBiono GeneTech Co., Ltd., Shenzhen, China]; in group three [p53 plus ultrasonic irradiation (p53+US) group], 10 ml Ad-p53 and 0.05 ml/kg ultrasound contrast agent (SonoVue^®^; Bracco, Milan, Italy) were infused through the microcatheter (1×10^11^ vp) under ultrasonic irradiation (using a Vivid 7 Ultrasound with an M3S probe operating at a frequency of 3.4 MHz and a mechanical index of 1.0; GE Healthcare, Freiburg, Germany). Postoperatively, the femoral artery was ligated and the incision was sutured.

### Determination of serum VEGF levels by enzyme-linked immunosorbent assay (ELISA)

Blood samples were collected from the ear vein one day prior to and four days after the Ad-p53 perfusion; the serum was separated by centrifugation and stored at −80°C. Serum VEGF levels were determined using a double-antibody sandwich assay, with the specific experimental procedures performed according to the manufacturer’s instructions (Senxiong Biotech Industry Co., Ltd., Shanghai, China). Following the termination of staining, the optical density (OD) values were detected using a microplate reader (Bioworld Technology, Nanjing, China) at a wavelength of 495 nm. The absolute value of serum VEGF was calculated according to the standard curve.

### Immunohistochemistry

On the fourth day after Ad-p53 infusion, the rabbits were anesthetized with 50 mg/kg pentobarbital sodium to obtain liver cancer specimens. Five liver specimens from each group were fixed in 4% paraformaldehyde and a routine paraffin-embedded method (19) was performed to obtain 5-μm serial sections. Immunohistochemistry was performed on the serial sections, which were stained for with monoclonal mouse anti-rabbit p53 (1:500; cat. no. ab2462), polyclonal rabbit anti-human MMP2 (1:500; cat. no. ab16033), monoclonal mouse anti-mouse VEGF (1:500; cat. no. ab1316) and monoclonal rabbit anti-human glyceraldehyde 3-phosphate dehydrogenase (1:500; cat. no. ab128915) antibodies, which were all purchased from Abcam (Cambridge, UK). Light microscopy was performed at ×400 magnification for each pathological chip in five randomly selected, unrepeated fields. Subsequently, the pathological image analysis system was used to measure the integral OD value of each field under the same conditions.

### Western blot

On the fourth day after Ad-p53 infusion, the remaining five liver specimens from each group were frozen in liquid nitrogen, ground and the total proteins were extracted using RIPA buffer (Thermo Fisher Scientific, Waltham, MA, USA) (containing protease inhibitors). Following SDS-PAGE gel electrophoresis, the total proteins were transferred to a polyvinylidene difluoride membrane, blocked with 5% skimmed milk and incubated with p53 antibody (dilution, 1:1000) at 4°C overnight. The total proteins were washed with Tris-buffered saline plus Tween-20 solution (Sigma-Aldrich, Munich, Germany), conjugated with horseradish peroxidase secondary antibody for one hour at room temperature, and stained using an enhanced chemiluminescence (ECL), fixation and development technique with an ECL substrate (cat. no. 32106; ECL, Thermo Fisher Scientific).

### Statistical analysis

SPSS statistical software (version 11.0) was used to perform the statistical analyses and all data are expressed as the mean ± standard error of the mean. Multi-group comparisons were performed using one-way analysis of variance, and the pre- and post-treatment data from each group was compared using the unpaired t-test. P<0.05 indicated a statistically significant difference.

## Results

### Effect of different Ad-p53 administration methods on the p53 expression

**Figure 1:**
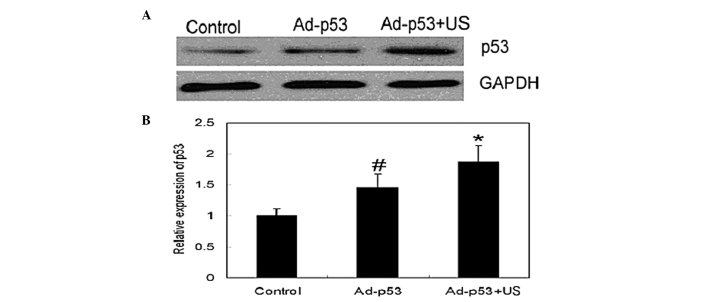
Effect of different administration methods on the protein expression levels of p53 in tumor tissues. (A) Representative western blot image; and (B) statistical results of the relative protein expression levels of p53. n=5; ^#^P<0.05, vs. the control group; ^*^P<0.05, vs. the Ad-p53 group. Ad-p53, adenovirus-p53; US, ultrasonic irradiation.

**Figure 2:**
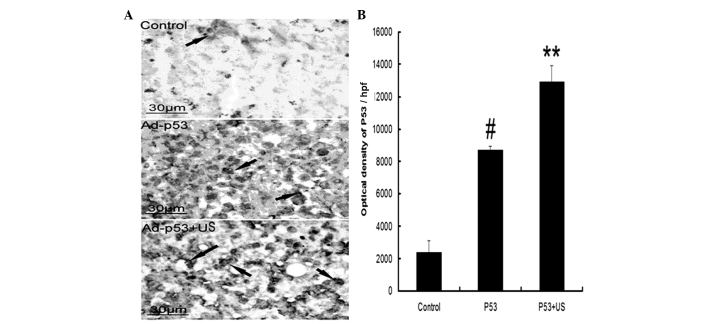
Effect of different administration methods on p53 protein expression levels in tumor tissues. (A) Representative immunohistochemical images in the control, p53 and p53+US groups (from top to bottom). Arrow, positive immunohistochemical staining for p53. (B) Integral optical density results calculated using the pathological image analysis system. n=5; ^#^P<0.05 and ^**^P<0.01, vs. the control group. Ad-p53, adenovirus-p53; US, ultrasonic irradiation.

To observe whether different administration methods would affect the transfection efficiency of Ad-p53, western blotting was used to detect the wt-p53 expression levels in the different groups (Fig. 1). The results demonstrated that p53 expression was higher in the p53 treatment group compared with the control group and the p53 expression was significantly higher in the p53+US group compared with in the p53 group, indicating that ultrasonic irradiation may improve the transfection and expression efficiency of Ad-p53. Additionally, to observe the morphological characteristics of p53 expression, immunohistochemistry was performed (Fig. 2); the results demonstrated that p53 was distributed throughout the cytoplasm. Furthermore, pathological image analysis techniques were used to determine that the p53-positive products (VEGF and MMP2) were most highly expressed in the p53+US group followed by the p53 group, while the control group exhibited the lowest expression.

### Effect of Ad-p53 therapy on serum VEGF levels

The growth and invasion of tumors is closely associated with the angiogenesis of tumor tissues. As the key nutritional factor in angiogenesis, VEGF is closely associated with tumor growth, invasion and metastasis; therefore, an ELISA was used to detect the serum levels of VEGF in the three groups (Table I). No significant difference was identified in the serum VEGF levels among the three groups prior to the treatment. Following Ad-p53 treatment, no significant difference was identified in the serum VEGF levels between the control and p53 group; however, the serum VEGF level in the p53+US group was significantly lower than in the control group.

### Effect of Ad-p53 therapy on the protein expression levels of MMP2 and VEGF

**Figure 3:**
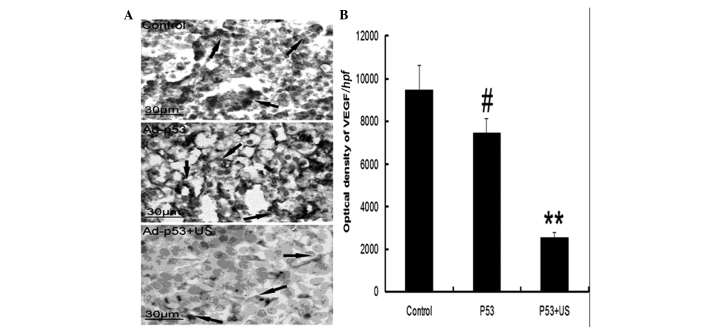
Effect of different Ad-p53 administration methods on MMP2 protein expression levels in tumor tissues. (A) Representative immunohistochemical images in the control, p53 and p53 + US groups. Arrow, positive immunohistochemical staining for MMP2. (B) Integral optical density results calculated using the pathological image analysis system. n=5; ^**^P<0.01, vs. the control group. Ad-p53, adenovirus-p53; MMP2, matrix metalloprotein 2; US, ultrasonic irradiation.

**Figure 4:**
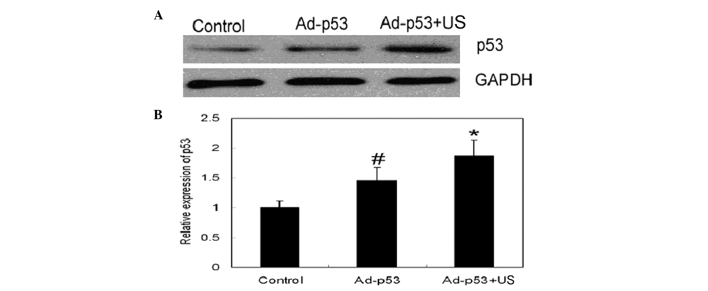
Effect of different Ad-p53 administration methods on VEGF protein expression levels in tumor tissues. (A) Representative immunohistochemical images in the control, p53 and p53+US groups. Arrow, positive immunohistochemical staining for VEGF. (B) Integral optical density was calculated using the pathological image analysis system. n=5; ^#^P<0.05 and ^**^P<0.01, vs. the control group. Ad-p53, adenovirus-p53; VEGF, vascular endothelial growth factor; US, ultrasonic irradiation.

The invasion and metastasis of tumor tissues involves the destruction of the basement membranes, which surround the tumor tissues. The MMP family is the predominant family of proteins involved in basement membrane destruction. Additionally, angiogenesis is considered to be closely associated with the invasion and metastasis of tumors. In the present study, combined with pathological image analysis, immunohistochemistry was performed to semi-quantitatively evaluate the protein expression levels of MMP2 and VEGF. The results demonstrated that MMP2 expression was significantly enhanced in tumor tissues (Fig. 3) and that Ad-p53 infusion therapy could partially alleviate the MMP2 expression level; although no statistically significant difference in MMP2 expression was demonstrated in the Ad-p53 group compared with the control group (P>0.05), the expression level of MMP2 in the p53+US group was significantly reduced compared with the control group (P<0.05). Similarly, VEGF expression levels were significantly enhanced in tumor tissues (Fig. 4) and p53 infusion therapy significantly reduced VEGF expression compared with the control group (P<0.05). In the p53+US group, the tumor tissue expression level of VEGF was significantly reduced compared with the control group (P<0.01).

## Discussion

To improve the expression level of the therapeutic p53 gene in tumor cells, the present study combined TAC and ultrasonic-irradiating therapeutic genes bearing ultrasound contrast agent. The results of the three groups of abovementioned animal experiments demonstrated that CIAIUI may significantly increase the expression of p53 protein in tumor tissues, as p53 protein expression levels were significantly higher in the p53+US group compared with the p53 and control groups. The results also demonstrated that tumor invasion and metastasis were associated with angiogenesis, and VEGF was the factor most closely associated with angiogenesis. As the majority of VEGF is secreted by the tumor cells, it may increase the permeability of the vascular walls (20,21). The present study identified that serum VEGF levels increased in a rabbit model and were highly expressed in tumor tissues; however, VEGF expression was reduced in the peripheral blood and tumor tissues of the p53 group, and significantly reduced in the p53+US group. This reduction in VEGF expression may be due to the high expression levels of p53 promoting tumor cell apoptosis, which reduces the excessive proliferation of tumor cells, therefore, decreasing VEGF expression levels. The present study also demonstrated that the invasion process was associated with the erosion and destruction of the basement membrane by tumor tissues. Previous studies have identified that the metastasis-associated protein family MMP is involved in this process, among which MMP2 was of the most importance (22,23). The present study identified that MMP2 protein expression levels were significantly increased in the control group, but downregulated in the Ad-p53 treatment groups. The expression of MMP2 was significantly downregulated in the p53+US group compared with the control group, which is consistent with the VEGF and wt-p53 expression patterns determined in the present study. The abovementioned results indicated that CIAIUI-administered Ad-p53 may significantly increase p53 expression levels in tumors.

Although VX2 hepatic carcinoma is an artificial metastatic squamous cell carcinoma that exhibits different biological characteristics from primary liver cancer, its advantages of stable biological characteristics and easy duplication mean that it is currently the most commonly used large animal cancer model (24,25). Therefore, VX2 cells were suitable for achieving the aim of the present study. In conclusion, CIAIUI via IIHTBA may significantly improve therapeutic gene expression levels within tumor cells. Furthermore, it is an efficient and safe method for targeted gene introduction, possesses advantages that are absent from traditional gene transfection methods and may provide basic understanding towards gene therapy in the future.

## Figures and Tables

**Table I:** Impact of different Ad-p53 administration methods on the serum VEGF expression levels before and after treatment.

	Serum VEGF expression level, (pg/ml) mean ± SEM	
	
Group (n=10)	Pre-treatment	Post-treatment
Control	396.96±85.95	559.44±10.90
Ad-p53	510.75±51.17	410.86±34.60*
Ad-p53 + US	404.71±37.09	224.14±15.41**

* P<0.05 and

** P<0.01, vs. the control group post-treatment.

VEGF, vascular endothelial growth factor; SEM, standard error of the mean; US, ultrasonic irradiation.
